# Nonlinear analysis by phase space reconstruction of healthy elderly voices

**DOI:** 10.1590/2317-1782/20232021280en

**Published:** 2023-06-26

**Authors:** Luana Alves Fernandes, Viviane Cristina de Castro Marino, Evelyn Alves Spazzapan, Débora Godoy Galdino, Lídia Cristina da Silva Teles, Arlindo Neto Montagnolli, Debora Sayuri Kakuda, Eliana Maria Gradin Fabbron

**Affiliations:** 1 Departamento de Fonoaudiologia, Faculdade de Filosofia e Ciências, Universidade Estadual Paulista “Júlio de Mesquita Filho” – UNESP - Marília (SP), Brasil.; 2 Departamento de Fonoaudiologia, Universidade do Unoeste Paulista – UNOESTE - Presidente Prudente (SP), Brasil.; 3 Departamento de Fonoaudiologia, Universidade de São Paulo – USP - Bauru (SP), Brasil.; 4 Universidade Federal de São Carlos – UFSCar, São Carlos (SP), Brasil.

**Keywords:** Voice, Voice Quality, Acoustic, Elderly, Nonlinear Dynamics

## Abstract

**Purpose:**

To compare the results of the non-linear acoustic analysis of elderly male and female voices, speakers of Brazilian Portuguese.

**Methods:**

Recordings of 14 men and 15 women were used. The voices were consensually judged to be vocally healthy by three trained speech therapists. The non-linear acoustic analysis was performed by the Phase Space Reconstruction (PSR) analysis using the Voice Analysis program.

**Results:**

A significant difference was observed in the parameter irregularity (p = 0.001) and spacing (p = 0.005), with worse results for the male group. While 93% of male voices presented degrees 2 or 3 of irregularity, these degrees were observed in 53% of female voices. In 78.6% of male voices, medium to large spacing was observed, a fact observed only in 26.7% of women.

**Conclusion:**

The results of the non-linear analysis, through the Phase Space Reconstruction, using the CIS Protocol, in the voices of the elderly, showed the best result in terms of the number of curves (four or more). Regarding the irregularity of the tracing, in men, the majority presented grades 2 and 3 and in women, half presented grade 1. Regarding the spacing, 78.6% of the male voices had medium to large spacing, a fact observed only in 26.7% of women.There was a difference between the sexes in the vocal findings of the elderly by the CIS protocol with the PSR, pointing out worse results irregularity and spacing in the male population, which suggests greater vocal aperiodicity in elderly men.

## INTRODUCTION

Senescence is a natural physiological process of aging that involves bodily changes in the elderly. As part of the human body, the larynx also undergoes natural changes called presbylarynx^(1)^. Aging can lead to several impacts on the body, such as soft tissue atrophy, cartilage ossification and cellular alterations and, as a consequence, it results in changes in the voice that can be inferred by the acoustic analysis of the voice signal^(2-5)^. As an inherent part of the aging process, the structural changes caused by presbyphonia can reflect on the vocal quality of the elderly^(1)^.

The increase in the elderly population and the search for treatments that improve the quality of life, resulted in an increase in the search for care for guidance and therapy for voice disorders by the elderly, which boosted research on the efficiency of voice therapies to overcome voice disorders in the elderly^(6-14)^.

Traditional acoustic measures, such as Fundamental Frequency (f_o_), jitter, shimmer and Noise-to-Harmonic Ratio (NHR), can differentiate genders and vocal deviation in the elderly^(2)^. In this context, previous studies have investigated vocal changes throughout life through acoustic measurements^(2-6)^. Regarding disturbance measures, a previous study showed higher values in the elderly, with this value being higher for men compared to women^(2)^. The information originating from these studies must be taken into account during the vocal assessment and therapeutic monitoring of the corresponding age group, including the elderly^(3)^.

The non-linear analysis of the sound signal represents another possibility of voice evaluation^(15)^. According to the literature, this type of analysis^(15)^ emerged in the 1990s, and some researchers in Brazil studied the non-linear voice analysis through visual patterns^(16-21)^. Also called recurrent quantification measure, this analysis was identified as an important complementary instrument in vocal analysis and with a promising future in studies to quantify a vocal deviation^(21)^.

As a type of non-linear analysis, which describes the vocal dynamics in a tracing, which can be viewed in a two- or three-dimensional way, depending on time, Phase Space Reconstruction (PSR) stands out among the other analyses.

The method for extracting measurements is carried out using the delay time technique, and allows capturing the vibrations of the vocal folds in relation to time^(22)^.

The PSR tracing, or also called trajectory, represents all the dynamics of a system, where periodic systems are represented by closed trajectories and aperiodic systems by irregular trajectories. A fundamental step necessary to obtain the PSR is the application of the delay time method, through a time delay vector. This delay is fundamental in measuring the phase space. The final graph formed from the phase space reconstruction represents the vibratory dynamics of the vocal folds in relation to time and its configuration is greatly influenced in relation to vocal quality. This method offers more information about the non-linear dynamics present in the human voice. PSR is an effective tool in differentiating normal and deviated voices, as changes directly affect phase space trajectories^(20)^.

It should be noted that there is no standardization in the terminology, in the way of measuring and in the description of grades for the three analyzed parameters of the two-dimensional graph. In this sense, a recent study was carried out with the aim of developing a proposal to standardize the analysis of the phase space reconstruction (PSR) tracing in healthy voices of adult women and men^(20)^. Standardization was performed using the CIE protocol (curve, irregularity and spacing) which evaluates the number of curves in the trajectory (0 to 4 or more, with the best result being 4 or more turns) and the parameters of irregularity and spacing from a numerical scale of 0 to 3 points, from best to worst configuration. The Voice Analysis software developed by Montagnoli was used for this study^(23)^. Using this same methodology^(20)^, a later study^(24)^ investigated the voices of patients with vocal fold pathologies (nodule, cyst and sulcus vocalis). The findings of this study showed a predominance of 4 curves and mild irregularity, since the spacing parameter was small for the presence of cysts and nodules, and medium for the presence of sulcus vocalis.

Although the qualitative analysis of the PSR tracings is a resource for diagnostic complementation in vocal assessment, this analysis is still being implemented and investigated and, therefore, studies aimed at standardizing the tracings in voices of different life cycles are essential.

The qualitative evaluation of the graphs generated with the PSR tracing, through the CIE protocol, is a resource that may help to further understand the voice quality of the elderly from the point of view of non-linear acoustic analysis. Given the vocal changes that may occur in the elderly, it is relevant to investigate how such changes can be inferred through non-linear analysis of the sound signal and, in particular, through the PSR with evaluation by the CIE protocol. In addition, it is also relevant to understand whether these measures differ in terms of sex. It is assumed that the values of the number of curves, the grade of irregularity and the grade of spacing of the PSR are different from the values found for adults using the CIE protocol. Furthermore, these characteristics are expected to be different between men and women.

In this context, this article aimed to analyze voices of elderly people through non-linear analysis by PSR with standardized CIE protocol and compare these findings between genders.

## METHODS

This study is part of a broader research that involved acoustic analysis (nasalance values and changes in vocal quality) in different life cycles through acoustic measures, which was approved by the Human Research Ethics Committee of the institution, under the Decisions No. 0657/2013 and 1,054,283/2015. All participants signed the Free Prior Informed consent. The study included audio voice recordings of elderly men and women, collected and stored between 2013 and 2017. Thus, this was an analytical and cross-sectional observational study.

### Case study

The study included 29 individuals, aged between 60 and 93 years, of both sexes without vocal complaints, 14 men (mean age 73.9 years) and 15 women (mean age 77.1 years). The inclusion criteria were as follows: participants of both sexes, aged sixty years or older, with satisfactory general health conditions on the day of the examination, and adequate vocal quality for age according to auditory-perceptual assessment.

This evaluation was carried out consensually by experienced three speech-language pathologists in the evaluation of speech and voice alterations, based on a perceptive-auditory evaluation using the G parameter of the GRBAS Scale^(25)^. As proposed by Yamasaki et al.^(26)^, this study considered the general grade of vocal quality equal to zero or one, as they indicate a normal variation in vocal quality.

The following individuals were excluded: smoker at the time of data collection or history of smoking in the last five years; professional vocal training; history of head and neck surgeries; history of neurological, pulmonary or respiratory diseases; speech-language pathology care for voice; vocal complaints in the week of the recording; individuals with hearing complaints and speech disorders; participants who reported nasal obstruction, cold and/or flu or respiratory allergies on the day of collection.

### Procedures

The voice recordings of the elderly, of both sexes, were selected from those previously stored in the Laboratório de Análise Articulatória e Acústica *[Laboratory of Articulatory and Acoustic Analysis]* of the institution.

### Sample recording and edition

The recordings were carried out in a room with acoustic treatment using a MARANTZ PMD660 digital recorder, configured for single channel recording, with a sampling rate of 44.1 kHz and 16 bits of resolution; using a e835 Sennheiser microphone fixed on a pedestal, at 45 grades and five centimeters away from the mouth of the participants.

The sample consisted of the sustained emission of the vowel /a/ in pitch and usual loudness according to the instructions given to the participants. The recordings of three vocal productions of each patient were performed. The choice of one of these three vocal emissions for use in the study was carried out by one of the researchers, who carried out a visual inspection of the narrow-band spectrographic tracing of the acoustic signal using the PRAAT software^(27)^ and thus determined the most stable emission.

All audios included in this study were analyzed as described and then used to extract the PSR in the Voice Analysis software^(24)^.

### Non-linear acoustic analysis

**Figure 1:**
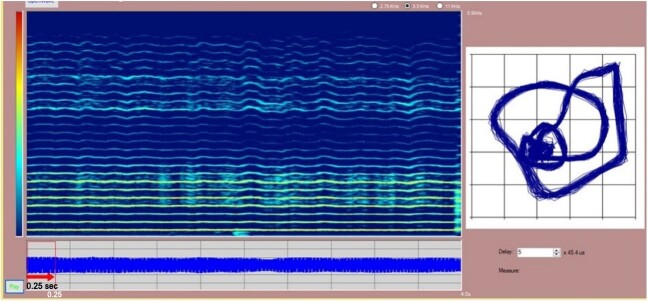
Print of the Voice Analysis software screen, with the spectrogram image and the PSR graph

Source: Galdino^(20)^

The non-linear analysis of the acoustic signal was performed using the Phase Space Reconstruction (PSR) method, whose image was extracted by the Voice Analysis software, provided by Montagnoli^(23)^. According to standardization^(20)^, the recordings were edited disregarding the first second of the acoustic signal and using the subsequent time of 0.5 seconds for men and 0.25 seconds for women (Figure 1).

Then, it is necessary to carry out the visual perceptual evaluation of the image from the graph generated by the software. In assessing the grade of spacing by the CIE protocol, using the software, the researchers selected and measured the distance in millimeters between the trajectories in the PSR image, taking into account the most frequent spacing of the tracing. It should be noted that the regions of the trajectories that contained the most frequent width were measured^(20)^. The delay time setting in the software for extracting measurements was automatically defined in the Voice Analysis software^(23)^ when the PSR graph was generated. After measuring the graph and marking the spacing size, a screenshot of the Voice Analysis software^(23)^ was taken, keeping only the trajectory graph. In this context, three speech-language pathologists were trained to perform the visual perceptual evaluation of the graphics image generated according to the CIE protocol.

The CIE Protocol^(23)^, in which curve measurements can vary from 0 to 4 curves or more, with the best result being the value of 4 or more curves, was used for the visual perceptual analysis of the generated graph. The irregularity of the wave trajectory can vary from grade 0 to 3; being grade 0: normal (mild sporadic), grade 1: mild (mild or moderately sporadic throughout the tracing), grade 2: Moderate (moderate in every stroke or severe in curve regions) and grade 3: Severe (severe in curve regions or sporadic).

The wave path spacing is measured by the software and categorized on a scale from 0 to 3 millimeters, with grade 0: Minimum (up to 5.9 mm in the tracing), grade 1: Small (from 6 mm to 8.9 mm), Grade 2: Medium (from 9 mm to 13.9 mm) and Grade 3: Large (greater than 14 mm)

**Figure 2:**
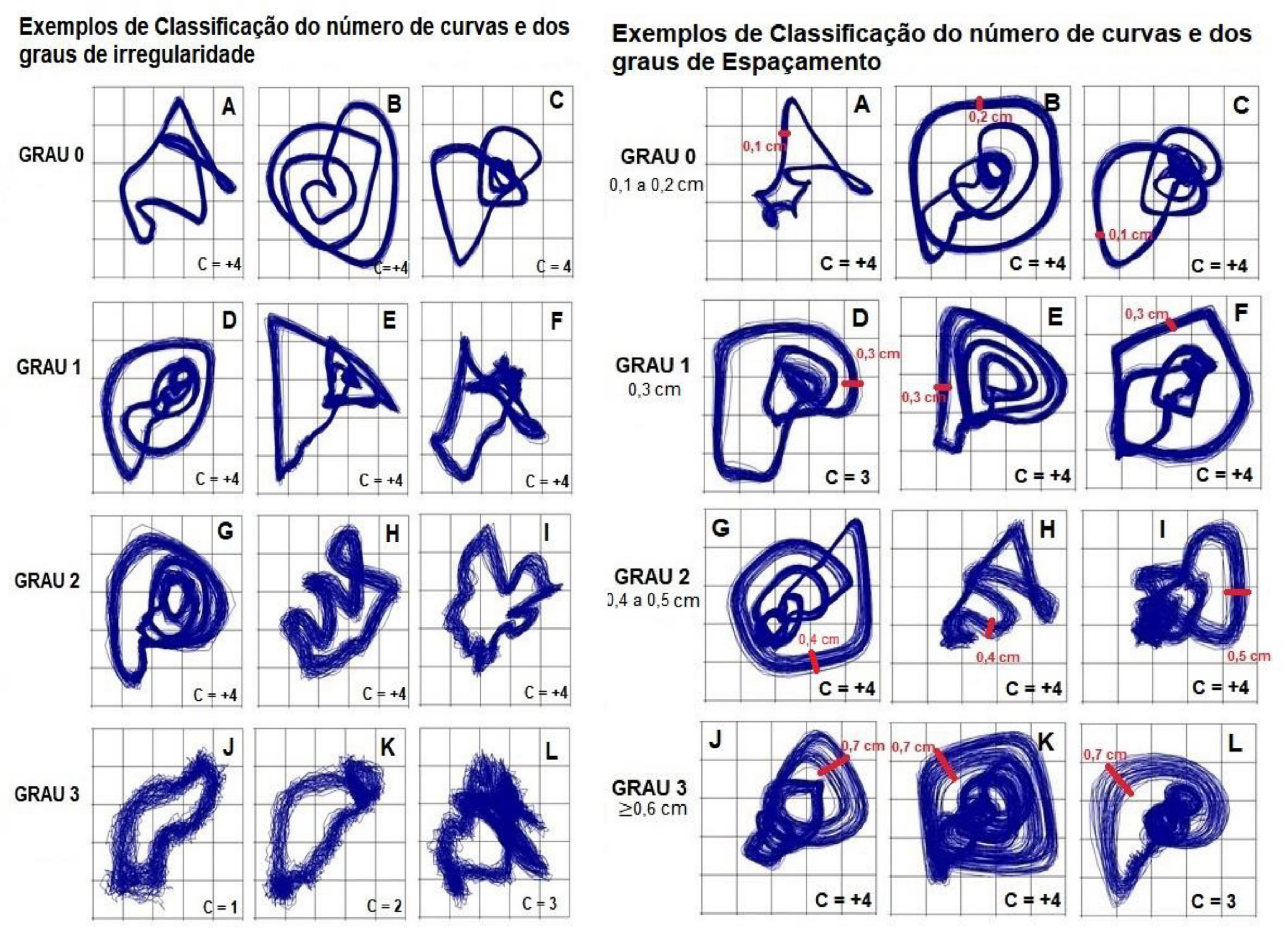
Example of classification of numbers and curves and grades of irregularity and spacing according to Galdino^(20)^

Source: prepared by the authors

The example of the number of curves and the grade of irregularity and spacing of the image, used in the proposal of the CIE Protocol^(23)^ according to Figure 2, was followed to carry out the visual perceptual evaluation of the generated images.

It should be noted that the number of curves can be identified in Figure 2 by observing the following characteristics: a) The number of turns is counted in the graphics of the PSR with a trajectory in a spiral format; b) The number of trajectory curves in phase space is counted in graphs with random formats; and c) In the case of completely chaotic trajectories, in which it is not possible to identify the curves, it is suggested to identify as “no defined curves”. With regard to the analysis of the grade of irregularity, the configuration of the tracing must be observed from the best to the worst, being 0 for regular lines throughout the course, 1 for irregular lines in only one portion, 2 for lines with more than one portion irregular, 3 for traces with slight irregularity throughout the course and 4 for the entire course with irregular traces. The software allowed selecting and measuring the image of the PSR graph, and the width of the most frequent spacing of the tracing. As described by Galdino, 2019, the regions of the trajectories that contained the most frequent width were measured^(23)^.

The visual judgment of the images, parameters, curves and irregularity was made by three trained speech-language pathologists, as proposed by Galdino^(20)^. It should be noted that this study used a newer version of the Voice Software (Montagnoli)^(23)^, in which it was possible to measure the spacing on the computer itself, right after extracting the PSR image. The images generated by the software were randomly organized and submitted to speech-language pathologists for analysis. The sending of 20% of the sample was replicated to carry out the agreement analysis.

The answer that was repeated by two evaluators was decided as the final answer and, when there was no agreement between them, a fourth judge, also trained, was used to determine the answer with greater precision.

### Data analysis

The inter and intra judge agreement of the visual perceptual analysis of the graphics extracted by the software was performed by the CCI test with results ranging from 0.91 to 1. However, the inter-judge agreement was 0.91 in the evaluation of the curves and 0.49 in the irregularity, which included the participation of a fourth evaluator, in situations where there was no agreement of at least two evaluators. The spacing value was extracted at the time of the measurement, and it was not necessary to perform the CCI test.

The Mann-Whitney U test with a significance level of p<0.05 was used to compare results between genders.

## RESULTS

Data are presented through graphs and tables derived from the non-linear analysis by PSR.

Regarding the voices of the elderly of both sexes, the results show that 100% of them had normal voice patterns, with a high number of curves in the trajectory and a better number of curves, that is, with four or more curves in the tracing^(23)^.

**Figure 3:**
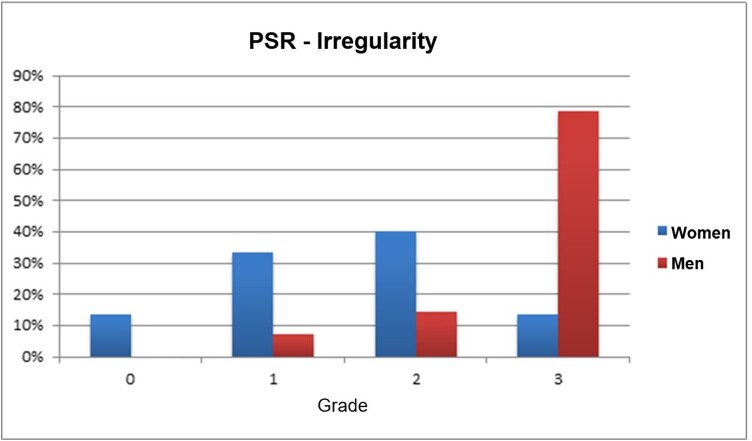
Classification of grades of irregularity of PSR tracings in percentage of men and women

Source: prepared by the authors

**Figure 4:**
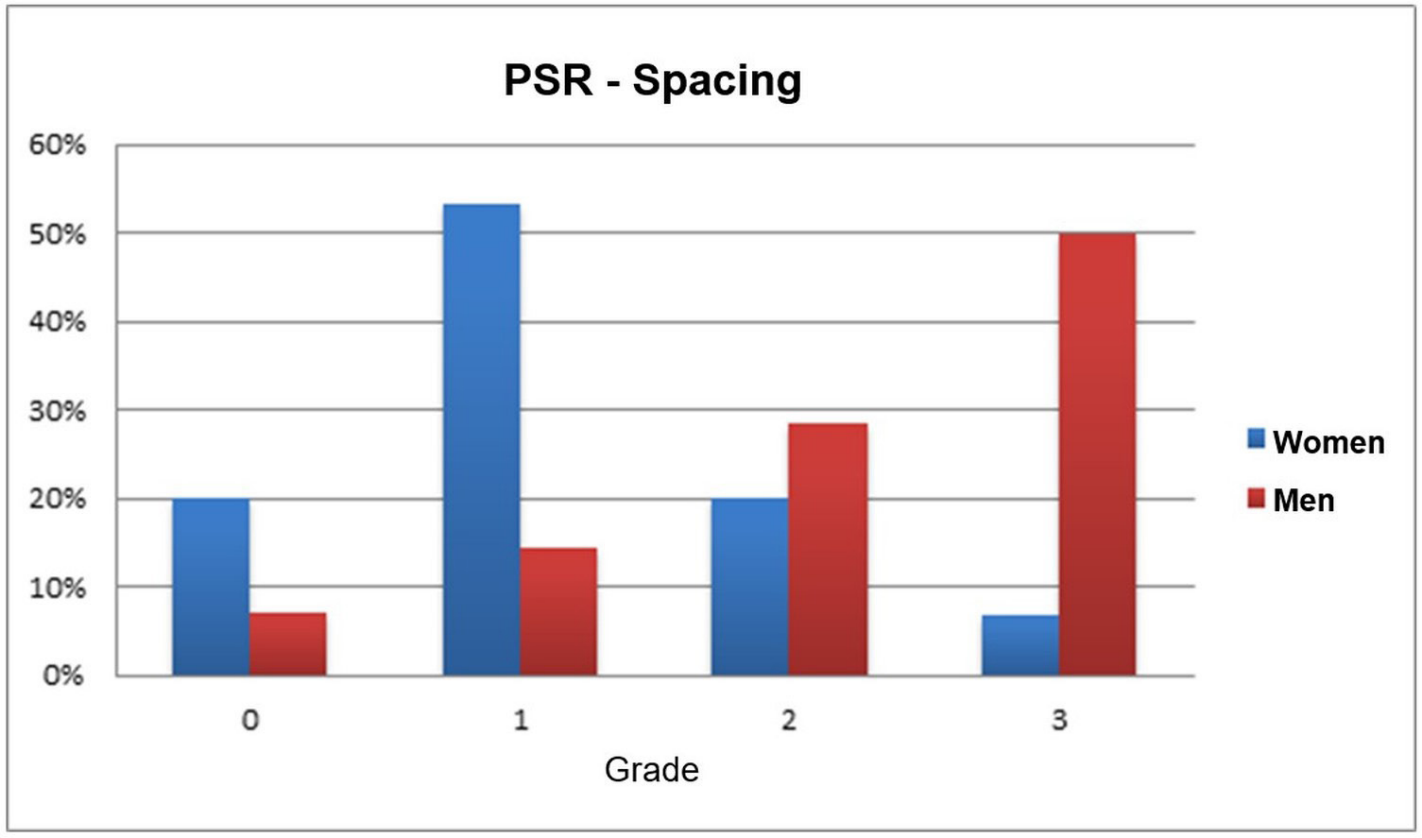
Classification of grades of spacing of PSR tracings in percentage of men and women

The grades referring to the irregularity and spacing parameters obtained by the analysis with the REF CIE protocol of male and female voices are shown in figure 3 and figure 4.

Table 1 shows the classification of grades of irregularity and spacing of tracings in the PSR of men and women in percentage.

**Table 1:** Inferential analysis of the variables curve, irregularity and spacing according to sex

Variable	Sex	Mean	SD	Mann-Whitney U test	Z	p-value
Curve	Female	4.00	0.00	105.000	0.000	1.000
	Male	4.00	0.00			
Irregularity	Female	1.53	0.92	31.500	-3.418	0.001*
	Male	2.71	0.61			
Spacing	Female	1.13	0.83	43.000	-2.817	0.005*
	Male	2.21	0.97			

Mann-Whitney U test

* p<0.05

**Caption:** SD=standard deviation.

Author: own file

While 93% of the male voices had grades 2 or 3 of irregularity, these grades were observed in 53% of the female voices. In turn, 78.6% of male voices had medium to large spacing, which was observed only in 26.7% of females.

## DISCUSSION

Non-linear acoustic analysis has been described as a way to analyze chaotic systems, such as vocal production.

Previous studies investigating the REF^(17,18)^ used the terms loops or loops as a way of analyzing the plot of the graph generated in the analysis extraction. The CIE (Curve, Irregularity and Spacing) protocol proposal discussed that, aiming at a better understanding and interpretation of the generated graph, instead of loops, the nomenclature should be replaced by curves, since when modifying the delay time in the program, the trajectory can be visualized in other angles and, in this way, it was possible to notice that the loops, in fact, are curves in the trajectory that overlap in the phase space. In this sense, the CIE protocol allowed understanding that the loops were actually curves that were superimposed, generated through the voice signal.

The results obtained by the CIE protocol regarding the number of voice curves showed that 100% of the subjects, both women and elderly men, had four or more curves in the trajectory of the PSR. The number of curves is related to better vocal quality, and voices with more curves suggest greater voice periodicity. Regarding women, the findings are in line with the study by Dajer et al.^(28)^, who found a number of loops of degrees 4 and 3 (referring to the CIE protocol curves) in adult women without laryngeal pathologies.

In this same study, the authors found worse loop degrees for women with Reinke's Edema.

The results presented in this study are also in line with the findings of Galdino^(20)^, who reported that the vast majority of participants in his study of adult men and women (18 to 50 years old) with healthy voices, without vocal complaints and history of laryngeal surgeries, had four or more curves using the CIE Protocol. Regarding the number of curves found in the voices of elderly men, the results can also be compared to those presented by Galdino^(20)^ for adult men with healthy voices.

The aforementioned non-linear analysis studies show that the number of curves changes according to the individual's vocal deviation. Thus, the number of curves found in this study is explained by the neutral vocal quality of the elderly participants.

Regarding the irregularity parameter of the PSR, 40% of elderly women with healthy voices were classified as grade 2 (moderate irregularity throughout the tracing or severe irregularity in curve regions), which indicates that the irregularity of the tracing can present with greater degree in the tracing in voices of elderly women evaluated by auditory-perceptual evaluation with degree less than or equal to 1. On the other hand, studies with healthy voices of young women reported maximum regularity, in opposition to the group of women with Reinke's Edema that presented the worst value of irregularity^(28)^. Tracing irregularity is related to the presence of noise in the voice signal, such as turbulent air flow from the lungs and conditions of the laryngeal mucosa, which are some of the factors considered as possible sources of noise in vocal dynamics. The possible increase in traditional acoustic measures, such as Jitter, Shimmer and signal-to-noise ratio, suggests the irregularity in the orbit of the tracing of the PSR^(17)^.

Vocal changes can be seen from the fourth decade of life, approximately in adults, even before the onset of menopause^(2,3)^. Such changes result from hormonal changes mainly in women, including the larynx. In addition, advancing age leads to alterations involving laryngeal structures, such as decreased neuromotor control, changes in the laryngeal mucosa, cartilage calcification, muscle atrophy, increased collagen fibers and decreased elastic fibers and hyaluronic acid, among other effects of presbylarynx, as prominence of the vocal process and laryngeal arching^(29)^.

Thus, these changes impact the results of acoustic measures, which must be considered in a vocal assessment. Although some studies report higher values in jitter, shimmer and NHR measurements in old age, non-linear acoustic measurements have gained prominence in research and vocal clinics due to their reliability, their relationship with auditory perceptual assessment and the possibility of analyzing more irregular and aperiodic acoustic signals where traditional analysis is not able to assess.

As for the irregularity parameter of the PSR of male voices, 50% of the elderly men were classified as grade 3 (severe, complete or sporadic). The irregularity of male voices investigated by Galdino^(20)^ was classified in 90.8% with grade 0 (normal). This difference can be explained by the study's proposal to assess the voices of adults who have lower vocal aperiodicity compared to the elderly. Laryngeal muscle aging and fusiform glottic cleft reflect changes in the voice quality of the elderly, making their voices more aperiodic and, therefore, with a greater grade of irregularity^(2-5)^.

Worse values of tracing irregularity were found in men when compared to the values found in women. This difference between the sexes may be a result of the asymmetry of vibration of the vocal folds that occurs in old age. And, as reported by other studies, this characteristic is observed to a greater degree in males^(29,30)^.

It should be noted that the acoustic study of elderly voices, through non-linear analysis, using the PSR is pioneering and there are no possible comparison studies. Thus, future research should be developed in order to better understand this analysis in healthy and dysphonic voices and also to understand the association between these measures and the auditory perceptual analysis. Furthermore, the existence of a number of curves compatible with normal voices, but with greater irregularity in the tracing, must be related to the fact that the elderly are vocally healthy. Thus, despite the presence of irregularity in the voice of the elderly, they continue to be considered with normal variation in vocal quality, according to the inclusion criteria of this study.

In voices with vocal deviation, the study by Dajer et al.^(17)^ considered tracing regularity between grades 5 and 0, with 5 being the most regular tracing and 0, the worst. Thus, the author reported that 75% of the voices were considered healthy, with grade 4; 18.75% had grades 5 and 6, and 25% had grade 3. In turn, most participants with Reinke's edema had grade 3, and those with vocal fold nodules had grade 2.

Different protocols for evaluating the regularity/irregularity of the tracing with different degrees of results are found in the literature, making it difficult to compare the results found. In this sense, and based on the proposal by Galdino^(20)^, the results found in this study show a moderate degree of irregularity.

Regarding the spacing parameter of the PSR of the participants in this study, the tracing of 53.33% of the elderly women with healthy voices was classified as grade 1 (small - from 6 mm to 8.9 mm in the trajectory). In addition, spacing was strongly related to disturbances in frequency and amplitude of voice signals, such as jitter and shimmer measurements^(20)^. These results are similar to the findings by Dajer et al.^(28)^ who found a high degree of regularity in voice recordings of 23 women aged 25 to 45 years. In this study, Dajer et al.^(28)^ used a four-degree scale with scores from 1 to 4; degree 4 being the best result, meaning trajectories with a high degree of convergence, that is, less spacing.

The spacing parameter of the PSR of the elderly in this study was classified as grade 3 (large = greater than or equal to 14mm of trajectory) in 50% of the voices of the healthy elderly. These findings are similar to those found by Galdino^(19)^ for the voices of adult men without vocal complaints through the visual patterns of vocal dynamics, who observed that 58.22% of the individuals were classified with a grade 3 spacing.

The vibration irregularity present in old age, more prominently in elderly men, could explain the difference between genders found in the spacing parameter, as also pointed out in another parameter of the traditional analysis, such as the shimmer^(30)^.

Different protocols for evaluating the spacing/convergence with different degrees of results are found in the literature, making it difficult to compare the results found. In addition, there are no studies in the literature with the population of elderly women. In this sense, and based on the proposal by Galdino^(20)^, the results found in this study show a mild degree spacing.

It is important to emphasize that the use of the Voice Analysis Software (Montagnoli)^(23)^ enabled and facilitated the analysis of the PSR. The following aspects were the main favorable points of using the program: easy handling; platform developed for Windows operating system; choice of the wave signal section according to the spectrography; and automatic measurement of delay time and spacing. The measurement is automatically calculated after selecting the section given in millimeters, offering the evaluator the best condition for graph analysis. Likewise, the use of the CIE^(20)^ protocol enabled the analysis of the PSR graphs.

### Limitation and recommendations

The limitations of the study are related to the small number of participants and the lack of otorhinolaryngological examination of the patients. None of the participants had vocal complaints and all of them were evaluated by auditory-perceptual assessment. In addition, future studies should compare the analysis of the PSR in different populations, of different age groups, for a better understanding of changes in this analysis with growth and aging.

## CONCLUSION

The findings of the non-linear analysis, through PSR, with the use of the CIE protocol, in elderly voices, showed the best result regarding the number of curves (four or more). Regarding tracing irregularity, the majority of men has grades 2 and 3, while half of the women had grade 1. Regarding spacing, 78.6% of male voices had medium to large spacing, which was observed only in 26.7% of females.

There was a difference between the sexes in the findings of acoustic measures of the voices of the elderly by the non-linear analysis using the PSR, with the use of the CIE Protocol, with the worst results being obtained in irregularity and spacing in the male population, which suggests greater vocal aperiodicity in elderly men.

Further studies are suggested with a larger elderly population, confronting auditory-perceptual measures that may better clarify the results described here.
