# Supplementary material for: Nonlinear analysis by phase space reconstruction of healthy elderly voices
Source: Codas. 2023 Jun 26;35(3):e20210280. doi: 10.1590/2317-1782/20232021280en (PMC10348666; doi:10.1590/2317-1782/20232021280en)
Supplement: Translation

# Translation: Análise não linear pela reconstrução do espaço de fase de vozes saudáveis de idosos

**DOI:** 10.1590/2317-1782/20232021280pt

**Authors:** Luana Alves Fernandes, Viviane Cristina de Castro Marino, Evelyn Alves Spazzapan, Débora Godoy Galdino, Lídia Cristina da Silva Teles, Arlindo Neto Montagnolli, Debora Sayuri Kakuda, Eliana Maria Gradin Fabbron

**Affiliations:** 1 Departamento de Fonoaudiologia, Faculdade de Filosofia e Ciências, Universidade Estadual Paulista “Júlio de Mesquita Filho” – UNESP - Marília (SP), Brasil.; 2 Departamento de Fonoaudiologia, Universidade do Unoeste Paulista – UNOESTE - Presidente Prudente (SP), Brasil.; 3 Departamento de Fonoaudiologia, Universidade de São Paulo – USP - Bauru (SP), Brasil.; 4 Universidade Federal de São Carlos – UFSCar, São Carlos (SP), Brasil.

**Keywords:** Voz, Qualidade da Voz, Acústica, Idoso, Dinâmica não Linear

## Abstract

**Objetivo:**

Verificar medidas acústicas das vozes de idosos pela análise não linear e realizar a comparação entre os sexos.

**Método:**

Participaram 29 idosos, falantes do Português Brasileiro, vocalmente saudáveis: 14 homens e 15 mulheres. Todas as vozes foram julgadas como vocalmente saudáveis por três fonoaudiólogas treinadas, por meio de avaliação perceptivo-auditiva. A análise acústica não linear foi realizada pela análise da Reconstrução de Espaço de Fase utilizando o programa Análise de Voz.

**Resultados:**

Foi observado diferença significativa no parâmetro irregularidade (p=0,001) e espaçamento (p=0,005) entre os sexos, com resultados piores no grupo masculino. Enquanto 93% das vozes masculinas apresentaram graus 2 ou 3 de irregularidade, estes graus foram observados em 53% das vozes femininas. Em 78,6% das vozes masculinas foi observado espaçamento de médio a grande, fato observado somente em 26,7% das mulheres.

**Conclusão:**

Os resultados da análise não linear, por meio da REF, com a utilização do protocolo CIE, em vozes de idosos, mostraram o melhor resultado quanto ao número de curvas (quatro ou mais). Em relação à irregularidade do traçado, nos homens, a maioria apresentou graus 2 e 3 e nas mulheres, a metade apresentou grau 1. Relativo ao espaçamento, 78,6% das vozes masculinas foi observado espaçamento de médio a grande, fato observado somente em 26,7% das mulheres. Houve diferença entre os sexos nos achados vocais de idosos pelo protocolo CIE com a análise de Reconstrução de Espaço de Fase, apontado piores resultados na irregularidade e espaçamento na população masculina, o que sugere maior aperiodicidade vocal em homens idosos.

## INTRODUÇÃO

O idoso sofre alterações corporais que fazem parte de um processo fisiológico natural do envelhecimento, denominado senescência. A laringe, como parte do corpo humano, também sofre modificações naturais denominadas de presbilaringe^(1)^. O impacto do envelhecimento provoca a atrofia dos tecidos moles, ossificação das cartilagens e alterações celulares e, como consequência, resulta em modificações na voz que podem ser inferidas pela análise acústica do sinal vocal^(2-5)^. A presbifonia é parte inerente do processo de envelhecimento, as mudanças estruturais podem refletir na qualidade vocal do idoso ^(1)^.

Por conta do crescimento da população idosa e a busca por tratamento que melhore a qualidade de vida, houve um aumento da busca, por parte dos idosos, por atendimento para orientação e terapia para o distúrbio vocal, o que impulsionou as pesquisas sobre a eficiência de terapias vocais para contornar o distúrbio da voz no idoso^(6-14)^.

As medidas acústicas tradicionais como Frequência Fundamental (f_o_), *jitter*, *shimmer* e *Noise- to-Harmonic Ratio* (NHR) têm capacidade de diferenciar os sexos e o desvio vocal nos idosos^(2)^. Estudos prévios verificaram as mudanças vocais ao longo da vida por meio de medidas acústicas^(2-6)^. Em relação às medidas de perturbação, estudo prévio apontou valores mais elevados em idosos, sendo esse valor maior para os homens comparado com o de mulheres^(2)^. As informações derivadas destes estudos devem ser levadas em conta durante a avaliação vocal e o monitoramento terapêutico da faixa etária correspondente, incluindo idosos^(3)^.

A análise não linear do sinal sonoro representa uma outra possibilidade de avaliação da voz^(15)^. Segundo a literatura, na década de 90 deu-se a inserção deste tipo de análise^(15)^, sendo que no Brasil alguns pesquisadores estudaram a análise não linear da voz por meio de padrões visuais^(16-21)^. Essa análise, também denominada de medida de quantificação recorrente, foi apontada com um instrumento complementar importante na análise vocal e com um futuro promissor nos estudos para quantificar um desvio vocal^(21)^.

A reconstrução do espaço de fase (REF) é um tipo de análise não linear, que destaca-se dentre as demais análises pois descreve a dinâmica vocal em um traçado, que pode ser visualizado de forma bi ou tridimensional, em função do tempo. O método para a extração de medidas é realizado por meio da técnica de tempo de atraso,e consegue captar as vibrações das pregas vocais em relação ao tempo^(22)^.

O traçado da REF ou também denominada trajetória representa toda a dinâmica de um sistema, onde sistemas periódicos são representados por trajetórias fechadas e sistemas aperiódicos por trajetórias irregulares. Para obtenção da REF, uma etapa fundamental necessária é a aplicação do método de tempo de atraso, por meio de um vetor de atraso de tempo. Tal atraso é fundamental na mensuração do espaço de fase. O gráfico final formado a partir da reconstrução do espaço de fase representa a dinâmica vibratória das pregas vocais em relação ao tempo e sua configuração sofre grande influência em relação à qualidade vocal. Este método oferece aos clínicos maiores informações sobre a dinâmica não linear presente na voz humana. A REF é uma ferramenta eficaz na diferenciação de vozes normais e desviadas, pois as alterações afetam diretamente as trajetórias do espaço de fase^(20)^.

Não há uma padronização na terminologia, na forma de mensurar e na descrição dos graus para os três parâmetros analisados do gráfico bidimensional. Recentemente, um estudo foi realizado com o intuito de desenvolver uma proposta de padronizar a análise do traçado da reconstrução do espaço de fase (REF) em vozes saudáveis de mulheres e homens adultos^(20)^. A padronização foi realizada por meio do protocolo CIE (curva, irregularidade e espaçamento) que avalia o número de curvas da trajetória (0 a 4 ou mais, sendo o melhor resultado, o valor de 4 ou mais voltas) e os parâmetros da irregularidade e espaçamento a partir de uma escala numérica de 0 a 3 pontos, da melhor para pior configuração. Para este estudo utilizou-se o programa de Análise de Voz desenvolvido por Montagnoli^(23)^. Utilizando esta mesma metodologia^(20)^, um estudo posterior^(24)^ investigou as vozes de pacientes com patologias na prega vocal (nódulo, cisto e sulco vocal). Os achados desse estudo mostraram predominância de 4 curvas e irregularidade de grau leve, já que para a presença de cisto e nódulos, o parâmetro de espaçamento foi pequeno e para sulco vocal, médio.

A análise qualitativa dos traçados da REF apresenta-se um recurso para complementação diagnóstica na avaliação vocal, entretanto, esta análise ainda está sendo implementada e estudada o que torna fundamental estudos que visem a padronização dos traçados nas vozes de diferentes ciclos de vida.

A avaliação qualitativa dos gráficos gerados com o traçado da REF, por meio do protocolo CIE, é um recurso que pode auxiliar a compreender, ainda mais, a qualidade vocal dos idosos, sob o ponto de vista da análise acústica não linear. Considerando as mudanças vocais que podem ocorrer em idosos, é de interesse verificar como tais mudanças podem ser inferidas por meio de análise não linear do sinal sonoro e, de modo particular, por meio da REF com avaliação pelo protocolo CIE. Também é de interesse verificar se essas medidas se diferenciam quanto ao sexo. Assume-se que os valores do número de curvas, do grau de irregularidade e do grau de espaçamento da REF são diferentes dos valores encontrados para adultos, utilizando o protocolo CIE. Também esperamos que estas características sejam distintas entre homens e mulheres.

O objetivo do trabalho foi analisar vozes de idosos por meio da análise não linear pela REF com protocolo padronizado CIE e comparar esses achados entre os sexos.

## MÉTODO

Este estudo é parte de uma pesquisa mais ampla que envolveu análise acústica (valores de nasalância e as mudanças da qualidade vocal) nos diferentes ciclos da vida por meio de medidas acústicas o qual foi aprovado pelo Comitê de Ética em Seres Humanos da instituição de origem, pareceres n^o^ 0657/2013 e n^o^ 1.054.283/2015. Todos os participantes assinaram o Termo de Consentimento Livre e Esclarecido. Foram utilizadas gravações de vozes em áudio pertencentes aos idosos, de ambos os sexos, coletadas e armazenadas entre os anos 2013 e 2017. Desta forma, o estudo desenvolvido foi do tipo observacional analítico e transversal.

### Casuística

Participaram deste estudo, 29 indivíduos, com idades entre 60 e 93 anos, de ambos sexos sem queixas vocais, 14 homens (média de 73,9 anos) e 15 mulheres (média de 77,1 anos). Os critérios de inclusão foram: participantes de ambos os sexos, com sessenta anos ou mais, com condições gerais de saúde satisfatórias no dia do exame, e qualidade vocal adequada para a idade de acordo com avaliação perceptivo-auditiva. Esta avaliação foi realizada consensualmente por três fonoaudiólogas com experiência na avaliação das alterações da fala e da voz, a partir de uma avaliação perceptivo-auditiva por meio do parâmetro G da escala GRBAS^(25)^. Foram considerados no presente estudo o grau geral da qualidade vocal igual a zero ou um por indicarem uma variação normal da qualidade vocal de acordo com Yamasaki et al.^(26)^.

Foram excluídos os indivíduos: fumante na ocasião da coleta de dados ou históricos de fumante nos últimos cinco anos; treinamento vocal profissional; histórico de cirurgias de cabeça e pescoço; histórico de doenças neurológicas, pulmonares ou respiratórias; tratamento fonoaudiológico para voz; queixas vocais na semana da gravação; indivíduos com queixas auditivas e alterações de fala; participantes que relataram obstrução nasal, resfriado e/ou gripe ou quadros alérgicos respiratórios no dia da coleta.

### Procedimentos

As gravações das vozes dos idosos, de ambos os sexos, foram selecionadas entre aquelas armazenadas previamente no Laboratório de Análise Articulatória e Acústica da instituição.

### Gravação e edição das amostras

As gravações foram realizadas em sala com tratamento acústico utilizando gravador digital MARANTZ modelo PMD660, configurado para gravação monocanal, com taxa de amostragem de 44.1 kHz e 16 bits de resolução; microfone Sennheiser modelo e835, fixado em um pedestal, posicionado a 45 graus e a cinco centímetros de distância da boca dos participantes.

A amostra foi constituída pela emissão sustentada da vogal /a/ em *pitch* e *loudness* habitual conforme orientação dada aos participantes. Foram realizadas as gravações de três emissões de cada paciente. A escolha de uma dessas três emissões vocais, para a continuidade da pesquisa, foi realizada por uma das pesquisadoras, que realizou a inspeção visual do traçado espectrográfico de faixa estreita do sinal acústico pelo *software* PRAAT^(27)^ e assim, determinava a emissão que se encontrava mais estável.

Todos os áudios que compuseram essa pesquisa foram analisados, conforme descrito e depois foram utilizados para a extração da REF no Programa Análise da Voz^(24)^.

### Análise acústica não linear

A análise não linear do sinal acústico foi realizada pelo método de Reconstrução do Espaço de Fase (REF), cuja imagem foi extraída pelo Programa Análise da Voz, cedido por Montagnoli^(23)^. Conforme padronização^(20)^, as gravações foram editadas com o descarte do primeiro segundo do sinal acústico utilizando o tempo subsequente de 0,5 segundos para homens e 0,25 segundos para mulheres (Figura 1).

**Figura 1:** Print da tela do programa de Análise de Voz, com a imagem de espectrograma e a do o gráfico REF; Delay Measure = Mensuração do Tempo de atraso

Fonte: Galdino^(20)^

A partir do gráfico gerado pelo programa é necessário a realização de avaliação perceptiva visual da imagem. Na avaliação do grau de espaçamento pelo protocolo CIE, com auxílio do próprio programa, foi selecionado e mensurado, em milímetros, na imagem da REF a distância entre as trajetórias, levando em consideração o espaçamento mais frequente do traçado. Ressalta- se que foram mensuradas as regiões das trajetórias que continham a largura mais frequente^(20)^. O tempo de atraso, que é uma configuração no programa para a extração das medidas, foi automaticamente definido no programa Análise de Voz^(23)^ ao ser gerado o gráfico da REF. Após a mensuração do gráfico e marcação do tamanho do espaçamento foi realizado o *print* do programa de Análise de Voz^(23)^ mantendo apenas o gráfico de trajetórias. Três fonoaudiólogos foram treinados para realizar a avaliação perceptiva visual da imagem dos gráficos gerada conforme o protocolo CIE.

Para a análise perceptiva visual do gráfico gerado foi utilizado o Protocolo CIE^(23)^. Neste protocolo, as medidas de curvas podem variar de 0 a 4 curvas ou mais, sendo o melhor resultado, o valor de 4 ou mais curvas. A irregularidade da trajetória da onda, pode variar de grau 0 a 3, sendo o grau 0: normal (leve esporádica), grau 1: Leve (leve em todo traçado ou moderada esporádica), grau 2: Moderado (moderada em todo traçado ou severa em regiões de curva) e grau 3: Severa (severa em regiões de curva ou esporádica). O espaçamento da trajetória de onda é mensurado pelo *software* e categorizado em uma escala de 0 à 3 milímetros, sendo o grau 0: Mínimo (até 5,9 mm na trajetória), grau 1: Pequeno (de 6 mm a 8,9 mm na trajetória), grau 2: Médio (de 9 mm a 13,9 mm na trajetória) e grau 3: Grande (maior igual a 14 mm da trajetória)

Para a realização da avaliação perceptiva visual das imagens geradas seguiu-se o exemplo de quantidade de curvas e de grau de irregularidade e espaçamento da imagem utilizada na proposta do Protocolo CIE^(23)^ de acordo com a Figura 2.

**Figura 2:** Exemplo de classificação do números e curvas e dos graus de irregularidade e espaçamento de acordo com Galdino^(20)^

Fonte: elaborado pelos autores

Ressalta-se que na Figura 2, o número de curvas pode ser identificada pela observação das seguintes características: a)Nos gráficos da REF com trajetória em formato espiralado conta-se o número de giros; b) Nos gráficos que apresentam formatos aleatórios, conta-se a quantidade de curvas da trajetória no espaço de fase; e c) No caso de trajetórias completamente caóticas, em que não é possível identificar as curvas, sugere-se que seja colocado “sem curvas definidas”. Para a análise do grau de irregularidade, deve ser observado a configuração do traçado; da melhor para pior, sendo 0 para traçados regulares em todo percurso, 1para traçados irregulares em apenas uma porção, 2 para traçados com mais de uma porção irregular, 3 para traçados com leve irregularidade em todo percurso e 4 para todo percurso com traçados irregulares. Com auxílio do *software*, foi possível selecionar e mensurar a imagem do gráfico da REF, a largura do espaçamento mais frequente do traçado. Ressalta-se que foram mensuradas as regiões das trajetórias que continham a largura mais frequente, conforme descrito em Galdino^(23)^.

O julgamento visual das imagens, dos parâmetros, curvas e irregularidade, foi feito por três fonoaudiólogas treinadas a partir dos modelos apresentados por Galdino^(20)^. Ressalta-se que na pesquisa ora relatada, foi utilizada uma versão mais nova do Programa de Voz^(23)^, em que foi possível medir o espaçamento no próprio computador, logo após a extração da imagem da REF. As imagens geradas pelo programa foram organizadas de forma aleatória e enviadas para as fonoaudiólogas. Para a realização da análise de concordância foi replicado o envio de 20% da amostra. Para a decisão de resposta, foi considerada aquela que se repetia por dois avaliadores e quando não havia uma concordância entre eles, foi utilizado um quarto juiz, também treinado, para determinar a resposta com maior precisão.

### Análise dos dados

A concordância inter e intra juiz da análise perceptiva visual dos gráficos extraídos pelo programa foi realizada pelo teste CCI com os resultados que variou de 0,91 a 1. Entretanto, a concordância interjuízes foi de 0,91 na avaliação das curvas e 0,49 na de irregularidade, e neste caso, houve a participação de um quarto avaliador, nas situações em que não houve a concordância de pelo menos dois avaliadores. O valor do espaçamento foi extraído no momento da realização da medida, não sendo necessário a realização do teste CCI.

Para a comparação dos resultados entre os sexos foi utilizado o teste não paramétrico Mann-Whitney com nível de significância p<0,05.

## RESULTADOS

Os dados são apresentados por meio de gráficos e tabelas derivados da análise não linear pela REF .

Em relação às vozes dos idosos de ambos os sexos, os resultados apontaram que 100% deles apresentaram padrões de vozes normais, com o elevada quantidade de curvas na trajetória melhor número de curvas, ou seja, com quatro ou mais curvas no traçado^(23)^.

Os graus referentes aos parâmetros da irregularidade e espaçamento obtidos pela análise com o protocolo CIE da REF das vozes masculinas e femininas são apresentados na Figura 3 e na Figura 4.

**Figura 3:** Classificação dos graus de irregularidade dos traçados na REF em porcentagem de homens e mulheres

Fonte: elaborado pelos autores

**Figura 4:** Classificação dos graus de espaçamento dos traçados na REF em porcentagem de homens e mulheres

A Tabela 1 apresenta em porcentagem a classificação dos graus de irregularidade e espaçamento dos traçados na REF de homens e mulheres.

**Tabela 1:** Análise inferencial das variáveis curva, irregularidade, espaçamento em função do sexo

Variável	Sexo	Média	DP	U de Mann-Whitney	Z	p-valor
CURVA	Feminino	4,00	0,00	105,000	0,000	1,000
	Masculino	4,00	0,00			
IRREGULARIDADE	Feminino	1,53	0,92	31,500	-3,418	0,001*
	Masculino	2,71	0,61			
ESPAÇAMENTO	Feminino	1,13	0,83	43,000	-2,817	0,005*
	Masculino	2,21	0,97			

Teste de Mann-Whitney

* p<0,05

**Legenda:** DP=desvio padrão

Autor: arquivo próprio

Enquanto 93% das vozes masculinas apresentou graus 2 ou 3 de irregularidade, estes graus foram observados em 53% das vozes femininas. Em 78,6% das vozes masculinas foi observado espaçamento de médio a grande, fato observado somente em 26,7% das mulheres.

## DISCUSSÃO

A análise acústica não linear tem sido descrita como uma forma de realizar análises de sistemas caóticos, como é o caso da produção vocal.

Estudos anteriores que pesquisaram a REF^(17,18)^ utilizaram os termos laços ou loops como uma forma de análise do traçado do gráfico gerado na extração da análise. Na proposta do protocolo CIE (Curva, Irregularidade e Espaçamento) discutiu-se que para maior compreensão e interpretação do gráfico gerado, ao invés de laços ou loops, a nomenclatura deveria ser substituída por curvas, (uma vez que ao modificar o tempo de atraso no programa, a trajetória pode ser visualizada em outros ângulos e com isso percebeu-se que os loops na verdade são curvas na trajetória que se sobrepõem no espaço de fase) já que o protocolo CIE permitiu a compreensão que os loops ou laços na realidade eram curvas que ficavam sobrepostas, geradas por meio do sinal da voz.

Os resultados obtidos pelo protocolo CIE quanto ao número de curvas das vozes revelaram que 100% dos sujeitos, tanto mulheres quanto homens idosos, apresentaram quatro curvas ou mais na trajetória da REF. A quantidade de curvas está relacionada a melhor qualidade vocal, vozes com mais curvas sugerem maior periodicidade da voz. Referente às mulheres, os resultados, foram compatíveis com o estudo de Dajer et al.^(28)^ que encontrou em mulheres adultas sem patologias laríngeas, o número de laços de graus 4 e 3 (nomenclatura referente às curvas do protocolo CIE). Neste mesmo estudo, os autores encontraram graus de laços piores para as mulheres com Edema de Reinke.

Os resultados aqui apresentados são comparáveis aos encontrados por Galdino^(20)^ que relatou que grande maioria das participantes do seu estudo de homens e mulheres adultos (18 a 50 anos) com vozes saudáveis, sem queixas vocais e antecedentes de cirurgias laríngeas, apresentaram quatro ou mais curvas utilizando o Protocolo CIE. Em relação ao número de curvas encontradas nas vozes de homens idosos, os resultados podem ser comparados aos apresentados por Galdino^(20)^ para homens adultos com vozes saudáveis.

Foi possível observar com os estudos de análise não linear citados, que a quantidade de curvas se altera conforme o desvio vocal do indivíduo. Desta forma, a quantidade de curvas encontradas no presente estudo se justifica pela qualidade vocal neutra dos idosos estudados.

Em relação ao parâmetro irregularidade da REF, 40% das mulheres idosas com vozes saudáveis, foram classificadas com grau 2 (irregularidade moderada em todo o traçado ou severa em regiões de curva), apontando que em vozes de mulheres idosas avaliada pela avaliação perceptivo-auditiva com grau menor ou igual a 1, a irregularidade do traçado pode apresentar-se com maior grau no traçado. Diferentemente, em estudos com vozes saudáveis de mulheres jovens, foi observada regularidade máxima em oposição ao grupo de mulheres com Edema de Reinke que apresentou o pior valor de irregularidade^(28)^. A irregularidade do traçado está relacionada com a presença de ruído no sinal da voz, o fluxo de ar turbulento proveniente dos pulmões e as condições da mucosa laríngea são alguns dos fatores considerados como possíveis fontes de ruído na dinâmica vocal. O possível aumento de medidas acústicas tradicionais, como o Jitter, Shimmer e relação sinal ruído sugerem a irregularidade na órbita do traçado da REF^(17)^ .

Mudanças vocais podem ser verificadas a partir da quarta década de vida, aproximadamente em adultos, antes mesmo do início da menopausa^(2,3)^. Tais mudanças resultam de modificações hormonais principalmente nas mulheres, incluindo a laringe. Além disso, com o avançar da idade, ocorrem alterações que envolvem as estruturas laríngeas como diminuição do controle neuromotor, alteração na mucosa laríngea, calcificação das cartilagens, atrofia muscular, aumento de fibras colágenas e diminuição de fibras elásticas e ácido hialurônico, entre outros efeitos da presbilaringe como proeminência do processo vocal e arqueamento laríngeo^(29)^.

Tais mudanças influenciam nos resultados de medidas acústicas, as quais devem ser consideradas numa avaliação vocal. Estudos apontaram maiores valores nas medidas de jitter, shimmer e NHR na terceira idade - entretanto medidas acústicas não lineares têm ganhado destaque na pesquisa e na clínica vocal devido sua confiabilidade, relação com a avaliação perceptiva auditiva e possibilidade de análise de sinais acústicos mais irregulares e aperiódicos onde a análise tradicional não é capaz de avaliar.

Relativo ao parâmetro de irregularidade da REF das vozes masculinas, 50% dos homens idosos, tiveram a classificação do grau 3 (severa completa ou esporádica). A irregularidade das vozes masculinas estudadas por Galdino^(20)^ foi classificada em 90,8% com grau 0 (normal). Essa diferença pode ser explicada pelo fato de o estudo propor a avaliação de vozes de indivíduos adultos que apresentam menor aperiodicidade vocal comparadas com idosos. O envelhecimento muscular laríngeo e fenda glótica do tipo fusiforme, refletem alterações na qualidade vocal do idoso tornando suas vozes mais aperiódicas e portanto com maior grau de irregularidade^(2-5)^.

Homens apresentam piores valores de irregularidade do traçado em relação às mulheres. Esta diferença entre sexos pode ocorrer devido a assimetria de vibração das pregas vocais que acontece na terceira idade. Esta característica é observada em maior grau no sexo masculino, conforme apontado em outros estudos^(29,30)^.

O estudo acústico de vozes de idosos, pela análise não linear, por meio da REF é pioneiro e não há estudos possíveis de comparações. Assim, futuras pesquisas devem ser desenvolvidas a fim de melhor compreender tal análise em vozes saudáveis e disfônicas e compreender a relação destas medidas com análise perceptiva auditiva. Além disso, o fato de haver quantidade de curvas compatíveis com vozes normais, mas apresentar irregularidade maior no traçado, deve-se ocorrer devido ao fato de os idosos serem vocalmente saudáveis. Assim, há irregularidade presente da voz do idoso, mas continuam sendo vozes consideradas com variação normal da qualidade vocal, de acordo com os critérios de inclusão deste estudo.

Em vozes com desvio vocal, o estudo de Dajer et al.^(17)^ considerou a regularidade no traçado entre os graus 5 e 0, sendo 5 o traçado com maior regularidade e 0, o pior. Foi encontrado que 75% das vozes consideradas saudáveis, apresentaram grau 4; 18,75% apresentaram grau 5 e 6,25% grau 3; Para os participantes com edema de Reinke, foi encontrado que a maioria apresentou grau 3 e que, aqueles com nódulos de pregas vocais, o grau 2.

A literatura apresenta diferentes protocolos de avaliação de regularidade/irregularidade do traçado com graus diversos de resultados, tornando difícil a comparação entre os resultados encontrados. Nesse sentido, os resultados encontrados neste trabalho apontam para uma irregularidade de grau moderado segundo a proposta de Galdino^(20)^.

Em relação ao parâmetro espaçamento da REF das participantes deste estudo, o traçado de 53,33% das mulheres idosas com vozes saudáveis foi classificado com grau 1 (pequeno – de 6 mm a 8,9 mm na trajetória).O espaçamento apresentou-se fortemente relacionado às perturbações de frequência e amplitude dos sinais da voz como às medidas de *jitter* e *shimmer* ^(20)^. Estes resultados se aproximam com aqueles encontrados por Dajer et al.^(28)^ que encontraram alto grau de regularidade em gravações de vozes de 23 mulheres de 25 a 45 anos de idade. No estudo de Dajer et al.^(28)^ foi utilizada em escala de quatro graus com os escores de 1 a 4; sendo o grau 4, o melhor resultado, significando trajetórias com alto grau de convergência, isto é, menor espaçamento.

O parâmetro de espaçamento da REF dos idosos do presente estudo foi classificado com grau 3 (grande = maior ou igual a 14mm da trajetória) em 50% das vozes dos idosos saudáveis. Tais achados são semelhantes ao encontrado por Galdino^(19)^ para vozes de homens adultos sem queixas vocais por meio dos padrões visuais de dinâmica vocal (PVDV), que observaram que 58,22% dos indivíduos foram classificados com espaçamento de grau 3.

A irregularidade de vibração presente na terceira idade, de forma mais proeminente em homens idosos, poderia justificar a diferença entre sexos encontradas no parâmetro espaçamento , como apontado também em outro parâmetro da análise tradicional, como shimmer^(30)^.

A literatura apresenta diferentes protocolos de avaliação de espaçamento/convergência com graus diversos de resultados, tornando difícil a comparação entre os resultados encontrados. Além disso, não há na literatura estudos com a população de mulheres idosas. Nesse sentido, os resultados encontrados neste trabalho apontam para espaçamento de grau pequeno segundo a proposta de Galdino^(20)^.

Importante ressaltar que o Programa Análise de Voz (Montagnoli)^(23)^ utilizado possibilitou e facilitou a análise da REF. Os principais pontos favoráveis da utilização do programa foram: fácil manuseio; plataforma desenvolvida para sistema operacional Windows; escolha do trecho de sinal de onda de acordo com a espectrografia; tempo de atraso e a mensuração do espaçamento dado automaticamente. A mensuração é calculada automaticamente após seleção do trecho dado em milímetros, oferecendo ao avaliador a melhor condição para análise do gráfico. Da mesma forma, o uso do protocolo CIE^(20)^, possibilitou a análise dos gráficos da REF.

### Limitações e recomendações

As limitações do estudo relacionam-se ao número pequeno de participantes e a ausência de exame otorrinolaringológico dos pacientes. Todos os participantes não apresentaram queixas vocais e foram avaliados pela avaliação perceptivo-auditiva. Além disso, estudos futuros devem comparar a análise da REF em diferentes populações, de diferentes faixas etárias, para melhor compreensão das mudanças desta análise com o crescimento e envelhecimento.

## CONCLUSÃO

Os resultados da análise não linear, por meio da REF, com a utilização do protocolo CIE, em vozes de idosos, mostraram o melhor resultado quanto ao número de curvas (quatro ou mais). Em relação à irregularidade do traçado, nos homens, a maioria apresentou graus 2 e 3 e nas mulheres, a metade apresentou grau 1. Relativo ao espaçamento, 78,6% das vozes masculinas foi observado espaçamento de médio a grande, fato observado somente em 26,7% das mulheres.

Houve diferença entre os sexos nos achados medidas acústicas das vozes de idosos pela análise não linear por meio da REF, com a utilização do Protocolo CIE, apontado piores resultados na irregularidade e espaçamento na população masculina, o que sugere maior aperiodicidade vocal em homens idosos. Estudos com uma população de idosos maior, confrontando com medidas perceptivo-auditivas poderão adicionar esclarecimentos aos resultados aqui descritos.
